# Rapidly Progressive Pulmonary Apical Fibrosis and Parenchymal Destruction in a Patient with Ankylosing Spondylitis

**DOI:** 10.1155/2020/8852515

**Published:** 2020-09-14

**Authors:** Hasan Ulusoy, Nazmiye Tibel Tuna, Aslı Tanrivermis Sayit

**Affiliations:** ^1^Division of Rheumatology, Department of Physical Medicine and Rehabilitation, Ondokuz Mayis University, Faculty of Medicine, Samsun, Turkey; ^2^Department of Pulmonary Medicine, Ondokuz Mayis University, Faculty of Medicine, Samsun, Turkey; ^3^Department of Radiology, Ondokuz Mayis University, Faculty of Medicine, Samsun, Turkey

## Abstract

Pulmonary apical fibrosis is a rare complication of ankylosing spondylitis (AS). The essential characteristics of this lesion are its very slow progression and frequently asymptomatic nature. Herein, we are presenting a patient with AS who rapidly developed pulmonary apical fibrosis in a 3-year period despite decreased musculoskeletal pains. The 60-year-old male applied with complaints of progressively increasing cough in the recent two years, dyspnea, and fatigue. He had no chronic disease except AS. He had no continuous medication except nonsteroid anti-inflammatory drugs for 2-3 days monthly since his musculoskeletal pains decreased in the recent years. His physical examination revealed reduced breath sounds in the upper zones of the right lung. Chest X-ray revealed increased diffuse opacity in the upper zones of the right lung. Thoracic high-resolution computed tomography showed a consolidation accompanied with traction bronchiectases compatible with chronic fibrosis in the upper lobe of the right lung. However, thoracic computed tomography of the patient performed 3 years ago did not reveal pulmonary apical fibrosis and parenchymal destruction. Biopsy revealed no finding of malignancy, granulomatous inflammation, or vasculitis. The results of cultures were negative. So, the patient was diagnosed as pulmonary involvement of AS, which developed in a 3-year period. This case has shown that extra-articular complications may continue to develop in patients with AS even if their musculoskeletal complaints have subsided. So, patients with AS should be followed up regularly with systemic examinations.

## 1. Introduction

Ankylosing spondylitis (AS) is a chronic inflammatory disease, and it mainly affects the axial skeleton and peripheral joints [1]. Extra-articular manifestations, such as ocular, cardiovascular, renal, and pulmonary involvement, may be observed in some of the patients with AS. The patients with extra-articular manifestations indicate higher disability and mortality rates. The most well-documented pulmonary manifestations of AS are apical pleural thickening seen in the advanced stage of the disease and formation of pulmonary apical fibrosis just beneath this thickening [2]. Pulmonary apical fibrosis is commonly asymptomatic and does not require a specific treatment [3]. However, it may be radiologically confused with tuberculosis and lung cancer. Inaccurate differential diagnosis may result in unnecessary tuberculosis therapy. In addition, secondary fungal and bacterial infections such as primarily *Aspergillus* may also develop in these lesions [4, 5]. Pulmonary apical fibrosis develops in about 2 decades and progresses very slowly [4, 6]. Herein, we are presenting a patient with AS who developed pulmonary apical fibrosis and parenchymal destruction in a 3-year period despite his musculoskeletal complaints being subsided. Our case is important to suggest that serious extra-articular complications of AS may develop also in the absence of musculoskeletal symptoms.

## 2. Case Report

**Figure 1:**
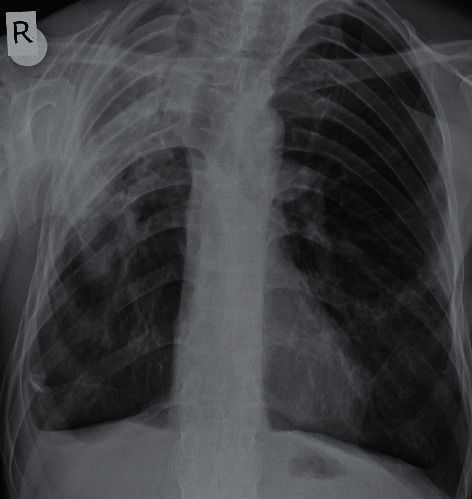
Chest X-ray shows diffuse opacities in the upper zone of the right lung. Also, it reveals deviation of the trachea to the right side and blunting of the right costophrenic sinus.

**Figure 2:**
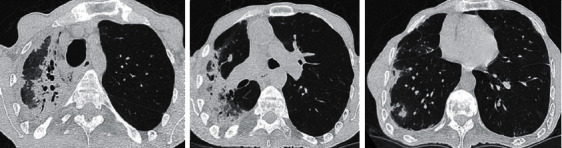
Thorax HRCT performed in 2019. It shows chronic consolidation with traction bronchiectasis compatible with fibrosis in the apical and posterior segments of the upper lobe which is extending to the superior segment of the lower lobe. Nodular alveolar densities in the lateral basal segment of the right lower lobe are also seen.

**Figure 3:**
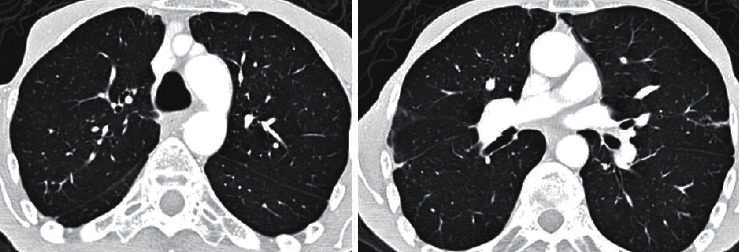
Thorax CT performed in 2016. There is no apical fibrotic changes and traction bronchiectasis in the upper zone of the right lung.

The 60-year-old male patient came to the chest diseases department of our hospital with the complaints of progressively increasing cough, dyspnea, and fatigue in the recent two years. The patient who denied hemoptysis said that he rarely produced sputum. He had no chronic disease except AS diagnosed in 1976. He never smoked. He had no alcohol consumption. He had no continuous medication except nonsteroid anti-inflammatory drugs (NSAIDs) for 2-3 days monthly since his pain of the musculoskeletal system decreased dramatically in the recent years. His physical examination revealed a blood pressure of 110/70 mmHg and 36.8°C body temperature. Heart beats were rhythmic, and no murmur was present. Breath sounds decreased in the upper zone of the right lung. The chest expansion and the lumbar Schober test measured 0 cm. Laboratory test results were found as follows: erythrocyte sedimentation rate (ESR) 72 mm/h, C-reactive protein (CRP) 28 mg/dL (normal range: 0–5), leukocytes 7800/mm^3^, hemoglobin 11 g/dL, platelets 323.000/mm^3^, and total protein/albumin ratio 6.8/3.3 g/dL. Other biochemical tests, electrolytes, and urinalysis results were within normal limits. Spirometric pulmonary function tests (PFT) revealed a restrictive pattern: a forced vital capacity (FVC) of 1.58 L (38% of predicted), a forced expiratory volume in 1 s (FEV1) of 1.50 L (46% of predicted), and an FEV1/FVC 94% and carbon monoxide diffusion capacity (DLCO) of 23 mL/min per mmHg (43% of predicted). Lung radiography revealed increased diffuse opacity in the upper zones of the right lung (Figure 1). Also, it showed right tracheal deviation and blunting of the right costophrenic sinus. Thoracic HRCT revealed the chronic consolidation with traction bronchiectasis compatible with fibrosis in the apical and posterior segments of the right upper lobe extending to the superior segment of the right lower lobe (Figure 2). In addition, there were also nodular alveolar densities in the lateral basal segment of the right lower lobe. Thoracic CT performed in 2016 showed mild bronchiectasis and sequela parenchymal bands in the middle lobe of the right lung. However, there was no apical fibrotic change, traction bronchiectasis, and parenchymal destruction in the upper lobe of the right lung (Figure 3). The comparison between thoracic HRCT and CT images suggested that pulmonary apical fibrosis and parenchymal destruction encountered in the right lung have developed in the recent three years. Gram staining, methylene blue stain, and Ehrlich–Ziehl–Neelsen (EZN) stain of the sputum revealed no pathogen, and all subsequent sputum cultures were negative. The tuberculin skin test resulted 0 mm, and the interferon-*γ* release assay (IGRA) detected no *Mycobacterium tuberculosis* infection. Bronchoscopy and transbronchial lung biopsy were performed. Biopsy did not reveal any finding of malignancy, granulomatous inflammation, or vasculitis. Lumbar vertebral and pelvis X-ray revealed bilateral grade IV sacroiliitis, syndesmophytes, and appearance of “bamboo spine.” As a conclusion, the patient was diagnosed with pulmonary involvement of AS including apical pleural thickening with upper lobe fibrosis and nonspecific interstitial abnormalities. Etanercept 50 mg/week and meloxicam 15 mg/day were administered. The patient was hospitalized for two weeks and included in the pulmonary rehabilitation programme. The next control examination after six weeks resulted as follows: ESR 36 mm/h, CRP 8 mg/dL, FVC of 41% of predicted, FEV1 of 49% of predicted, FEV1/FVC 96%, and DLCO 71% of predicted. After a six-month course of etanercept treatment, the patient did not develop new pulmonary lesion, and the drug was well tolerated without any adverse events.

## 3. Discussion

Pulmonary apical fibrosis is a rare extra-articular manifestation which is seen in the late stages of AS. The essential characteristics of this lesion which may be unilateral or bilateral are its very slow progression and frequently asymptomatic nature. The lesions may start to appear as linear or patchy opacities in the lung graphy and become larger by joining together in time. They may radiologically mimic tuberculosis and malignancy. Our case is important with respect to development of pulmonary apical fibrosis, parenchymal destruction, and various nonspecific interstitial abnormalities within three years despite musculoskeletal symptoms significantly reduced by years. The samples of transbronchial biopsy revealed no finding of malignancy, granulomatous inflammation, or vasculitis. The staining and culture tests from the obtained samples showed negative results. Also, PCR and IGRA tests performed for tuberculosis resulted negative. In the light of these results, the patient was diagnosed with pulmonary involvement of AS.

Pulmonary involvement was considered as a very rarely seen late-term complication of AS before the introduction of HRCT to the use. A retrospective analysis of lung radiographies from a large series of AS patients revealed 28 (1.3%) of 2080 patients, and most of those (25 patients) were reported to have upper lobe fibrosis. The studies which were conducted using HRCT in the later years have noted that various pleuroparenchymal manifestations were detected in 40–90% of the patients with AS [3, 7–9]. In these studies, AS was not typically associated with a pattern of a classic interstitial lung disease. HRCT findings in the AS patients are characterized with pulmonary apical fibrosis with or without cavitation, pleural thickening, parenchymal bands, septal thickening, bronchiectasis, emphysema, small airway diseases, ground glass attenuation, and pleural effusion. In a systematic review of 10 studies (303 patients), the prevalence of pleuroparenchymal manifestations was encountered to be 61% (185 patients) by HRCT in the AS patients. Upper lobe fibrosis, emphysema, bronchiectasis, ground glass attenuation, and nonspecific interstitial abnormalities were observed in 21 (6.9%), 55 (18.1%), 33 (10.8%), 34 (11.2%), and 101 (33%) patients, respectively [6]. The development of usual interstitial pneumonia with honey combing in the lower-middle zones of some cases was reported. However, pulmonary apical fibrosis differs from the pattern of classical usual interstitial pneumonia by leading to the development of extreme cicatrization, cavitation, and architectural distortion [10, 11].

In contrast with the conclusion in the previous years, abnormal HRCT findings are frequently (64–71%) encountered also in the early-stage AS patients (disease duration < 10 years) with normal lung graphy [12–14]. However, clinical importance of these abnormal findings encountered by HRCT is not yet known. Impaired pulmonary function tests (PFTs) are more rare (20–57%) although high rates (40–90%) of pleuroparenchymal abnormalities have been detected in the studies. In addition, the AS patients with and without pleuroparenchymal abnormalities have presented similar rates of impaired PFTs [12, 13, 15, 16]. Restrictive lung disease has been reported as the most common finding detected by PFT in patients with AS. Restrictive lung disease observed in patients with AS is associated with chest cage rigidity and reduced spinal mobility rather than pulmonary parenchymal disease [17]. Although the consideration that smoking causes interstitial inflammation and pulmonary parenchymal destruction is widely accepted, smoker and nonsmoker AS patients presented similar rates of abnormal HRCT findings [9, 12, 18, 19]. Turetschek et al. have reported the incidence of abnormal HRCT finding as 71% in the nonsmoker AS patients [14].

Since the causes of pulmonary parenchymal destruction in the AS patients are not yet clarified, no definitive treatment recommendation is available. It is considered that interstitial inflammation triggered by disease-specific mechanisms also plays a role, as well as chest cage rigidity. Chronic inflammatory cell infiltrates and prominent interstitial fibrosis have been reported in the biopsies [2, 18, 20, 21]. Therefore, reduction in the chest cage rigidity and suppression of systemic inflammation appear reasonable. It is known that pulmonary physiotherapy provides partial recovery in the restrictive destructions due to chest cage rigidity [2]. The effect of anti-TNF agents with proven efficacy in the AS patients with resistant axial and peripheral arthritis on the pulmonary involvements has not been widely investigated [22]. In a recent study, 27 of 89 AS patients (30.3%) received anti-TNF treatment; however, no association was detected between anti-TNF treatment and HRCT findings or PFT results [19]. Bargagli et al. have reported a beneficial effect of infliximab in the treatment of interstitial lung disease associated with rheumatoid arthritis [23]. On the other hand, these drugs may cause serious interstitial lung diseases [24].

Pulmonary apical fibrosis is a well-known extra-articular manifestation of AS, and it may be seen in the late stages of the disease. This manifestation is commonly asymptomatic and progresses very slowly in the years. However, the comparison between two separate CT imaging studies performed in our patient with an interval of three years revealed the development of rapidly progressive pulmonary apical fibrosis and parenchymal destruction. Because serious extra-articular complications may develop, patients with AS should be followed up regularly with systemic examinations even if their complaints have subsided.

## Data Availability

All data in this case report are taken from the clinical records.
